# Characterization and Source Apportionment Analysis of PM_2.5_ and Ozone Pollution over Fenwei Plain, China: Insights from PM_2.5_ Component and VOC Observations

**DOI:** 10.3390/toxics13020123

**Published:** 2025-02-06

**Authors:** Litian Xu, Bo Wang, Ying Wang, Huipeng Zhang, Danni Xu, Yibing Zhao, Kaihui Zhao

**Affiliations:** 1 Yunnan Key Laboratory of Meteorological Disasters and Climate Resources in the Greater Mekong Subregion, Yunnan University, Kunming 650091, China; 2 Xianyang Environmental Monitoring Station, Xianyang 712000, China; 3 Xianyang Meteorological Bureau, Xianyang 712000, China; 4 Information School, Yunnan University of Finance and Economics, Kunming 650221, China

**Keywords:** PM_2.5_ and O_3_, source apportionment, circulation classification, positive matrix factorization, Fenwei Plain

## Abstract

PM_2.5_ and volatile organic compounds (VOCs) have been identified as the primary air pollutants affecting the Fenwei Plain (FWP), necessitating urgent measures to improve its air quality. To gain a deeper understanding of the formation mechanisms of these pollutants, this study employed various methods such as HYSPLIT, PCT, and PMF for analysis. Our results indicate that the FWP is primarily impacted by PM_2.5_ from the southern Shaanxi air mass and the northwestern air mass during winter. In contrast, during summer, it is mainly influenced by O_3_ originating from the southern air mass. Specifically, high-pressure fronts are the dominant weather pattern affecting PM_2.5_ pollution in the FWP, while high-pressure backs predominately O_3_ pollution. Regarding the sources of PM_2.5_, secondary nitrates, vehicle exhausts, and secondary sulfates are major contributors. As for volatile organic compounds, liquefied petroleum gas sources, vehicle exhausts, solvent usage, and industrial emissions are the primary sources. This study holds crucial scientific significance in enhancing the regional joint prevention and control mechanism for PM_2.5_ and O_3_ pollution, and it provides scientific support for formulating effective strategies for air pollution prevention and control.

## 1. Introduction

Understanding the sources of PM_2.5_ and ground-level O_3_ is a crucial prerequisite for formulating effective prevention and control strategies. Prior research has indicated that atmospheric pollution displays pronounced regional characteristics. This is because pollutant concentrations are affected not only by local emission sources, but also by the cross-regional transport of pollutants. Merely utilizing observational techniques is inadequate for accurately identifying the impact of the long-distance transport of pollutants on the causation and contribution to pollution weather events [1,2,3]. Implementing methods such as the hybrid single-particle Lagrangian integrated trajectory (HYSPLIT), potential source contribution function (PSCF), and concentration weighted trajectory (CWT) analyses to conduct long-term quantitative studies on the trajectories and sources of pollutant transport can offer novel insights into regional pollution distribution characteristics, source apportionment, and causality analysis, enabling more effective strategies for air quality management and policy formulation [4,5,6,7].

Atmospheric pollution is closely linked to meteorological conditions. For instance, stable atmospheric conditions can lead to the accumulation of pollutants, while hot and dry weather exacerbates O_3_ pollution. Additionally, suitable wind patterns can transport pollutants generated in upwind cities to downwind areas. Furthermore, humidity was found to influence pollutant concentrations, with higher humidity levels generally leading to lower pollution levels due to increased particle deposition and chemical reactions [8,9,10]. Alternatively, atmospheric pollution is linked to large-scale circulation, which modulate meteorological conditions [11,12]. Variations in meteorological conditions have the potential to exacerbate atmospheric pollution and influence the effectiveness of emission reduction measures [13,14,15]. Several researchers have investigated the correlation between large-scale circulation patterns and pollutant concentrations. The occurrence of high-pressure weather systems is found to be associated with elevated levels of PM_2.5_ and O_3_ pollution [12,16], which in turn affects human health [17,18]. High-pressure systems are mainly associated with high temperatures, clear skies, and plentiful sunlight that are favorable for photochemical O_3_ reactions, as was observed in the south European, Yangtze River Delta (YRD), Pearl River Delta (PRD), and North China Plain (NCP) regions [19,20]. Regional pollutant transport, particularly from upwind industrial areas and biomass burning sources, plays a significant role in exacerbating PM_2.5_ and O_3_ pollution in the FWP. Large-scale meteorological patterns, such as prevailing winds and high-pressure systems, further facilitate the long-distance transport of pollutants to the region. Therefore, it is critical to reveal the inherent connection between atmospheric pollution and large-scale circulation.

Since the implementation of the Air Pollution Prevention and Control Action Plan (APPCAP) in 2013, primary PM_2.5_ pollution in China has been largely mitigated as a result of a series of strict PM_2.5_-focused strategies. Therefore, attention should now be shifted more towards the control of secondary PM_2.5_. PM_2.5_ and O_3_ co-control can be achieved by the synergistic reduction in VOC and nitrogen oxides (NOx) emission, as both are precursors of O_3_ and secondary PM_2.5_ [21,22]. These substances undergo a series of intricate chemical reactions, ultimately transforming into PM_2.5_ and O_3_ [23,24,25]. Research indicates that there exist significant disparities in industrial emissions across different time zones and regions. In addition to industrial emissions and vehicle exhausts, sources of PM_2.5_ precursors include agricultural activities and biomass burning, which generate substantial quantities of particulate matter and VOCs [26]. Similarly, in addition to contributions from industrial production and transportation, daily activities such as home renovation and paint application are significant sources of VOCs and NOx as precursors of O_3_ [27,28]. The primary receptor models currently employed for the source apportionment of PM_2.5_ and O_3_ include positive matrix factorization (PMF), principal component analysis (PCA), and chemical mass balance (CMB) [29,30,31,32]. Consequently, to gain a more comprehensive and accurate understanding of the formation mechanisms and pollution sources of PM_2.5_ and O_3_, future research should be conducted with greater depth of analysis and investigation in regions such as the FWP.

**Figure 1:**
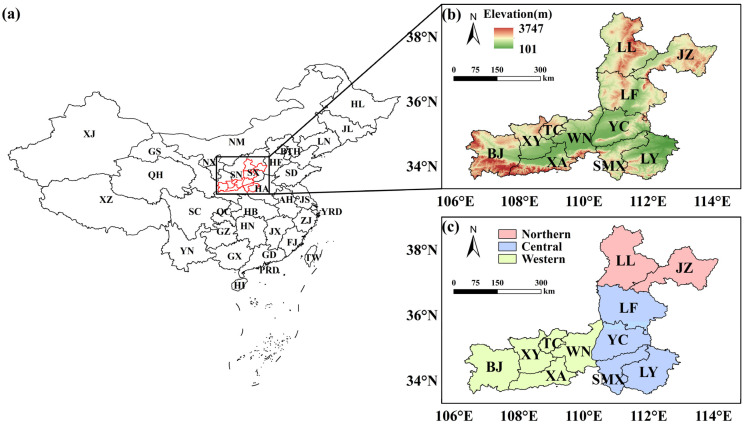
(**a**) Location map of the FWP; (**b**) topographic map of FWP; (**c**) schematic diagrams of three areas within the FWP.

The FWP, located at the intersection of three provinces, is characterized by its narrow geographical structure, which hinders the diffusion of pollutants (Figure 1, the map was created using ArcGIS Desktop software (version 10.8.2, Esri lnc., Redlands, CA, USA)). From 2015 to 2020, the FWP recorded an average PM_2.5_ concentration of 165 μg·m^−3^. In the summers of 2019–2021, the average O_3_ concentration peaked at 145.0 μg·m^−3^, more than doubling the winter levels [33,34]. According to the *Report on the State of the Ecology and Environment in China 2022*, the average proportion of good air quality days in the 11 cities of this region is only 62.5%, far below the national average of 86.5% [35]. Existing research predominantly provides general descriptions and data analyses of atmospheric pollution issues, lacking in-depth exploration of specific pollution sources and influencing factors. These studies largely focused on general pollution levels and source attribution and often overlooked the complex and intrinsic interactions between local pollution and large-scale circulation. Additionally, due to a lack of PM_2.5_ and VOC component observational data, VOC source apportionment still remains largely uncertain, which hinders the development of effective ozone control strategies [36,37,38,39]. Hence, there is an urgent need to intensify research efforts into the atmospheric pollution formation mechanisms in this area to inform the development of targeted pollution control policies based on scientific evidence.

In this study, to address the gap in the literature and provide insights into effective control strategies in the FWP, we applied HYSPLIT, circulation classification, and PMF, along with multi-source datasets. The objectives of this study were (1) to identify the atmospheric particulate matter and VOC components, (2) identify the potential source areas of PM_2.5_ and O_3_ by utilizing the HYSPLIT and large-scale circulation classification, demonstrating the underlying relationship between large-scale circulation and PM_2.5_ and O_3_ pollution, and (3) quantify the contributions of local industries as potential sources of PM_2.5_ and VOCs by employing the PMF. By examining the complex interactions between local emissions and large-scale circulation patterns, a large-scale circulation-based dynamic PM_2.5_ and O_3_ control philosophy can be adopted for FWP, which would be favorable for reducing both the peak PM_2.5_ and O_3_ levels more effectively.

## 2. Materials and Methods

### 2.1. Data Sources

#### 2.1.1. Observational Data

Hourly surface observations of pollutants such as PM_2.5_, O_3_, PM_10_, NO_2_, SO_2_, and CO were collected synchronously at 11 sites across the FWP regions during 2015–2022 using data obtained from the China National Environmental Monitoring Centre (CNEMC, https://www.cnemc.cn/, accessed on 20 December 2023). Data on PM_2.5_ and VOC components were obtained from the Shaanxi Environmental Monitoring Center Station (https://sthjt.shaanxi.gov.cn/, accessed on 12 February 2024), with monitoring conducted at the Wenxian Square in Xianyang. These data were obtained through standard environmental sampling methods, including gas chromatography and mass spectrometry for VOCs and ion chromatography for the water-soluble ions in PM_2.5_. Data quality control was performed in collaboration with the local air quality monitoring station, with anomalous values removed and linear interpolation applied to fill in missing data [40,41]. As per the Technical Regulation on Ambient Air Quality Index (on trial) (HJ 633-2012) [42] and Ambient Air Quality Standards (GB 3095-2012) [43], an O_3_ pollution day is defined as a day when daily maximum 8 h average O_3_ (MDA8 O_3_) surpasses 100 μg·m^−3^. PM_2.5_ pollution day is defined as a day when the daily average PM_2.5_ concentration exceeds 75 μg·m^−3^.

#### 2.1.2. Reanalysis Data

The data used for modeling weather related to high pollution in the FWP were obtained from the European Centre for Medium-Range Weather Forecasts (ECMWF) Global Atmospheric Reanalysis Data Generation 5 (ERA-5). The dataset primarily includes sea level pressure and 10 m horizontal winds, with a spatial resolution of 0.25° × 0.25°. Four daily time periods were selected, namely 00:00, 06:00, 12:00, and 18:00 UTC. The study region ranged from 80° to 130° E and 20° to 60° N. The required meteorological field data for the backward trajectory model consists of the National Centers for Environmental Prediction (NCEP) Global Data Assimilation System (GDAS, https://www.ready.noaa.gov/data/archives/gdas1/, accessed on 15 January 2024) meteorological data from 2015 to 2022. These data have a spatial resolution of 1° × 1°.

### 2.2. Methods

#### 2.2.1. Hybrid Single-Particle Lagrangian Integrated Trajectory (HYSPLIT)

The HYSPLIT model was applied to elucidate the potential sources of atmospheric pollutants in different cities over the FWP. HYSPLIT, jointly developed by the National Oceanic and Atmospheric Administration (NOAA) and the Australian Bureau of Meteorology, is a widely utilized system in regional pollution research. In this study, the HYSPLIT model within the TrajStat software framework (version 1.4.9, http://www.meteothink.org/downloads/index.html, accessed on 12 February 2024) was employed alongside meteorological data from the GDAS to investigate air transport trajectories of three representative cities situated across distinct regions of the FWP during both winter and summer seasons spanning the period from 2015 to 2022. Three representative cities, XA (34.27° N, 108.95° E), JZ (37.21° N, 112.17° E), and LY (34.62° N, 112.46° E), were selected as the terminal points for model simulations or particle reception points. The initial altitude for backward trajectory calculation was set at 500 m. The simulation of pollution periods, covering both winter and summer seasons, included the calculation of 72 h backward air mass trajectories arriving at these three simulation endpoints at 00:00, 06:00, 12:00, and 18:00 each day. In this study, we employed the Euclidean distance algorithm for the cluster analysis of these backward trajectories. Several studies have demonstrated that HYSPLIT can accurately track the sources of O_3_ and PM_2.5_ in key cities of the FWP and conduct cluster analysis [4,44,45,46,47]. Additionally, the PSCF and CWT methods were applied to identify major source regions contributing to O_3_ precursors. Details of these methods can be found in the Supplementary Materials.

Due much to their higher pollutant concentrations, and distinct meteorological and geographical characteristics, the three representative cities of XA, JZ, and LY were selected to identify the potential source area.

#### 2.2.2. Positive Matrix Factorization (PMF)

To provide scientific support for the formulation of effective PM_2.5_ and O_3_ control strategies for the FWP, the PMF method was used to identify the source factor and quantify the source contributions of PM_2.5_ and VOCs [29,48,49]. The fundamental principle of PMF is based on the decomposition of the pollutant content matrix *X_ij_* into the source component matrix *F_kj_* and the source contribution matrix *G_ik_*. The residual matrix *Q* represents the discrepancy between the actual data *X_ij_* and the analyzed data obtained from the decomposed matrices *F_kj_* and *G_ik_* (Equation (1)). To minimize the residual *Q*, both the factor matrix and the contribution matrix are subject to non-negative constraints. The mathematical foundation of this process can be expressed as follows:

(1) $$X_{ij}=\sum_{k=1}^{p}G_{ik}F_{kj}+E_{ij}$$

(2) $$Q=\sum_{i=1}^{n}\sum_{j=1}^{m}{\left[\frac{E_{ij}}{U_{ij}}\right]}^{2}$$

where *X_ij_* represents the concentration of the *j*-th sample, *G_ik_* denotes the contribution of the *k*-th factor to the source of the *i*-th sample, and *F_kj_* stands for the factor concentration of the *j*-th species in the *k*-th factor. *E_ij_* represents the residual concentration of every data value, while *U_ij_* signifies the uncertainty associated with each data point. Equation (3) serves as the basis for calculating this uncertainty. When the sample concentration falls below the method detection limit (*MDL*), a specific equation is employed to determine the uncertainty. This equation takes into account the characteristics of the analytical method and the nature of the samples, ensuring the accurate representation of the uncertainty in such cases. By incorporating this uncertainty estimation into the PMF model, we can obtain more robust and reliable results.

(3) $$U_{ij}=\frac{5}{6}\times MDL$$

When the concentration of sample was greater than the *MDL*, the following equation was used:

(4) $$U_{ij}=\sqrt{{\left(EF\times concentration\right)}^{2}\times {\left(0.5\times MDL\right)}^{2}}$$

The error fraction (*EF*) is typically determined empirically and generally ranges from 0.05 to 0.20 [50,51], depending on the quality and resolution of the available data. In this study, we used 0.20 for the *EF*, which is consistent with prior studies in similar regions [29,52], where uncertainties in both observational and model data are taken into account. This value provides a reasonable balance between model performance and observational error, particularly in the context of the available data in the FWP. Additionally, a set of studies have successfully applied the PMF model to identify key industries contributing to PM_2.5_ and O_3_ pollution [53,54,55,56]. Hence, we believe that the PMF model can be employed to track PM_2.5_ and O_3_ and quantify the relative contribution from each source.

#### 2.2.3. Circulation Classification

To investigate the underlying mechanism behind the long-range transport, a circulation classification method was applied to identify the dominated large circulation during PM_2.5_ and O_3_ pollution events. In the present study, we employed the T-model principal component analysis using oblique rotation, which was implemented in the weather classification software, cost733class-1.4. In this study, the criterion for classification was based on the sea level pressure observed on days with PM_2.5_ and O_3_ pollution. The geographical scope for this classification ranged from 80° E to 130° E longitudinally and from 20° N to 60° N latitudinally. Sea level pressure maps and 10 m wind field diagrams for each identified weather pattern were meticulously constructed at four specific times within a 24 h period: 00:00, 06:00, 12:00, and 18:00 UTC.

In the current study, the PCT algorithm, as implemented within the weather typing software cost733class-1.4, was utilized. Huth’s comprehensive evaluation of the algorithms incorporated in cost733class-1.4 revealed that the T-model obliquely rotated principal component analysis (PCT) algorithm demonstrates notable consistency and superior classification stability [57]. This makes it an efficacious tool for analyzing long-term variations in meteorological elements. The underlying principle involves representing the original data matrix *Z* as a product of the principal component matrix *F* and the loading matrix *A*, such that *Z = FA^T^*. Here, the dimensions of *Z* are *i × j*, those of *F* are *i × m*, and those of *A* are *j × m*, with *i* denoting the number of spatial grid points and *m* and *j* representing the number of temporal grid points. The loadings correspond to the eigenvectors of the original data’s correlation matrix, derived from the square roots of the associated eigenvalues. Principal components are ordered by the magnitude of their eigenvalues, with the leading components usually dictating the classification count. The first *N* principal components, characterized by larger eigenvalues, are retained for in-depth analysis. Their corresponding loading matrices *A* undergo diagonal rotation along with the principal components, culminating in the classification of weather conditions at each time interval based on the magnitude of the loads [58]. Owing to the relative stability of the PCT method, adjustments in the classification target do not precipitate significant alterations, thereby rendering the spatio-temporal field more consistently. Enhanced meteorological data accuracy and an expanded temporal series are anticipated to further augment the precision of this method. Moreover, the longer the temporal scale of the large-scale circulation under analysis, the more pronounced the advantages of the PCT method, resulting in more comprehensive and credible circulation classification outcomes [59]. PCT was chosen for circulation classification because it effectively captures the variability in large-scale circulation patterns in complex terrains like the FWP. Compared to other methods, such as k-means clustering or hierarchical clustering, PCT provides more stable results and better represents the correlation between meteorological variables, making it more suitable for the analysis of atmospheric circulation in the region [60,61]. Consequently, the PCT methodology emerges as an objective large-scale circulation classification approach, particularly suited for the FWP ’s complex terrain.

## 3. Results and Discussion

### 3.1. Concentration and Composition of PM_2.5_ and VOCs in the FWP

**Figure 2:**
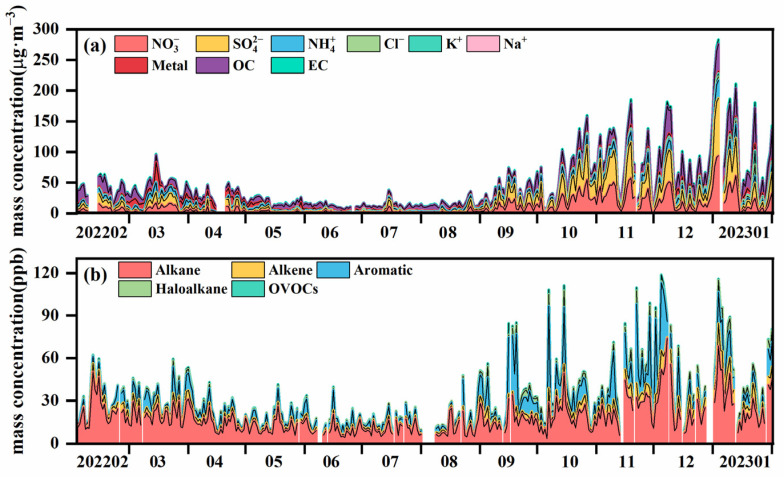
Concentration and composition of (**a**) PM_2.5_ and (**b**) VOCs.

As shown in Figure S1, the major air pollutants affecting the FWP are PM_2.5_ and O_3_, with PM_2.5_ concentrations peaking in winter and O_3_ concentrations peaking in summer. Additionally, there are significant differences in the spatial distribution of different air pollutants (Figure S2). Areas with high PM_2.5_ concentration were identified in the middle and west of the FWP, including LF, LY, and XA. O_3_ hotspot areas are primarily observed in the center of the FWP, specifically in YC and LY (more details are provided in the Supplementary Materials). To gain a deeper understanding of the sources of these pollutants in this region, we conducted a detailed analysis of the PM_2.5_ and VOC compositions in the representative city of XY in the FWP. The results are presented in Figure 2. The findings of this study indicate that there is a discernible pattern in the concentration of PM_2.5_, with higher levels observed during the winter months and lower levels during the summer months. The primary components of PM_2.5_ are water-soluble inorganic salts and organic compounds, which account for 64.45% and 26.83%, respectively. Metal elements exhibit an increased proportion in the spring. Among the water-soluble inorganic salts, the percentage of nitrates in summer is significantly lower than in winter. This phenomenon can be attributed to the thermal decomposition of nitrates due to elevated temperatures, which subsequently enters the gas phase through gas particle partitioning. Conversely, the concentration of sulfates increases in summer, primarily due to the enhanced solar radiation, which promotes the formation of oxidants such as O_3_ and OH radicals in the atmosphere. This intensifies secondary chemical processes, resulting in increased sulfate formation. Furthermore, the abundance of summer vegetation and elevated emissions of biogenic volatile organic compounds (BVOCs) facilitate the formation of secondary organic aerosols (SOAs) through atmospheric chemical processes such as photooxidation, thereby augmenting the proportion of total organic compounds. In this study, we observed that PM_2.5_ components exhibit clear seasonal variations, with higher concentrations of nitrates and sulfates in the winter. Such a significant enhancement is mainly associated with intense coal combustion and the use of motor vehicles. It is worth noting that the use of fireworks during winter is also a significant source of SOAs. These trends align with findings from similar climatic regions, such as Beijing, YRD, and Los Angeles [62,63,64].

Similarly, the concentration of VOCs exhibits a comparable seasonal trend to that of PM_2.5_, with higher levels observed in winter and lower levels in summer. This is primarily due to the substantial increase in VOC emissions from combustion sources related to winter heating, coupled with unfavorable meteorological conditions for pollutant dispersion. Among the various VOCs, alkanes constitute the highest mass concentration, accounting for 53.83%. The next most prevalent group of VOCs is that of aromatic hydrocarbons, which account for 26.23% of the total. This is followed by alkene (11.06%), halogenated hydrocarbons (7.20%), and oxygenated volatile organic compounds (OVOCs) (1.68%). However, the proportion of alkene increases during the winter months, potentially due to the reduced photochemical reactions caused by low temperatures and weak radiation, which leads to a decrease in the consumption of reactive VOC components, such as alkene.

### 3.2. Identification of Potential Source Areas for PM_2.5_ and O_3_ Pollution

Due to their extended photochemical lifetimes, PM_2.5_ and O_3_ can be transported hundreds or even thousands of kilometers in the atmosphere. Driven by local and regional atmospheric circulation, pollutants emitted in upwind cities are carried to downwind cities, thereby increasing the concentrations of PM_2.5_ and O_3_ in the downwind urban areas. Therefore, it is critical to elucidate the potential sources of atmospheric pollutants in different cities within the FWP.

#### Contributions from Potential Source Areas

**Figure 3:**
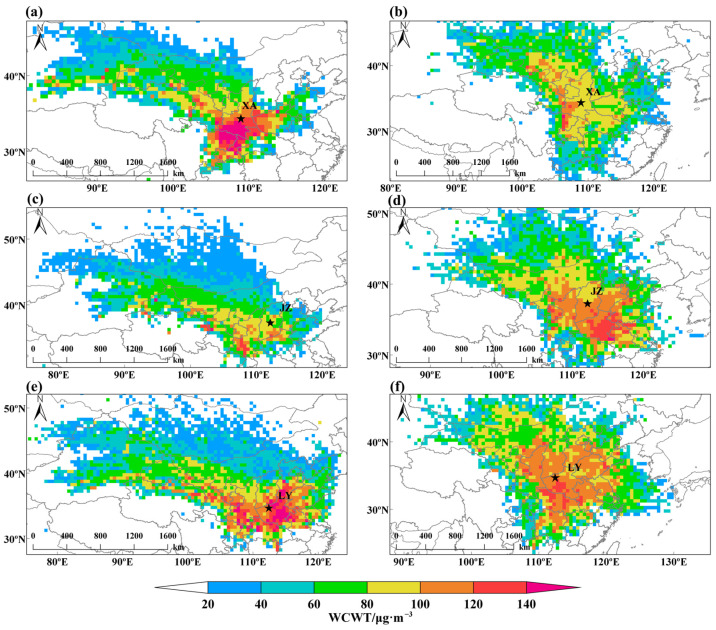
The weighted analysis results of urban pollutant concentrations in (**a**,**c**,**e**) winter and (**b**,**d**,**f**) summer seasons from 2015 to 2022. The map was created using MeteoInfo software (version 3.7.9, http://meteothink.org/, accessed on 12 February 2024). MeteoInfo is widely used for creating weighted analysis maps with the HYSPLIT model.

As discussed in the Supplementary Materials, the results of the HYSPLIT analysis indicate that, during winter, the FWP is primarily impacted by PM_2.5_ originating from the southern Shaanxi air mass and the northwestern air mass. However, in summer, it is mainly influenced by O_3_ originating from the southern air mass (Tables S1 and S2 and Figure S3). To further determine the contribution concentration of pollution source areas, the CWT method was adopted to quantify the grid-averaged concentration weights of pollution source areas. Figure 3 displays the trajectory pollution weights of PM_2.5_ and O_3_ concentrations of three representative cities in the FWP. We also conducted a PSCF analysis on the representative compositions, and further details are provided in the Supplementary Materials (Figure S5). The results of the weighted concentration weighted trajectory (WCWT) exhibit spatial distribution characteristics similar to those of the weighted potential source contribution function (WPSCF).

As depicted in Figure 3a, the northwestern regions encompassing GS, Mongolia, and the eastern part of XJ serve as potential source areas for PM_2.5_ pollution, with contributing concentrations ranging from 20 to 100 μg·m^−3^. These areas are particularly vulnerable to sand and dust pollution as well as desertification, further intensifying PM_2.5_ pollution. Neighboring regions, such as the southern SN, HA, CQ, and the eastern SC, contribute over 120 μg·m^−3^ to the PM_2.5_ concentration in XA. Pollution source areas affecting JZ (Figure 3c) are situated in the southern SN, GS, and HA, contributing to concentrations exceeding 100 μg·m^−3^. In contrast to XA and JZ, the potential source areas of PM_2.5_ in LY (Figure 3e) exhibit a patchy distribution, primarily attributed to the flat terrain of LY, which facilitates the transportation of pollutants from the surrounding regions. Regional pollutant transport, particularly from upwind industrial areas and biomass burning sources, plays a significant role in exacerbating PM_2.5_ and O_3_ pollution in the FWP. As shown in Figure S4, airmass passing through areas with intensive emissions can transport PM_2.5_, O_3_, and their precursors emitted from upwind sources to downwind regions. The important role of regional transport underscores the importance of strengthening the regional synergistic emission reduction strategy to mitigate ambient PM_2.5_ and O_3_ over the FWP. Additionally, the varied impact of regional transport from different source areas should be considered when formulating anthropogenic emission control strategies to effectively mitigate PM_2.5_ pollution.

Figure 3b illustrates the analysis of O_3_ pollution concentration weightings in representative cities during summer. The high values of WCWT are primarily concentrated in representative urban areas and gradually decrease towards the surrounding regions. During the summer, NX, GS, and Mongolia experience a climate characterized by high temperatures, low humidity, and high radiation, providing favorable meteorological conditions for O_3_ photochemical reactions. This contributes up to 20–100 μg·m^−3^ to the O_3_ concentration in representative cities and affects the FWP through the transport of air masses. The western area of XA and the eastern area of SC in the southwestern region contribute to O_3_ concentrations of over 100 μg·m^−3^ in the city, making it the main potential source of pollution. The O_3_ concentration in JZ (Figure 3d) is affected by the southern part of the city and the eastern part of HA, which contributes more than 100 μg·m^−3^ and 120 μg·m^−3^, respectively. The potential source areas affecting the O_3_ contribution of more than 100 μg·m^−3^ in LY (Figure 3e) are located in SN, SX, SD, HE, and HB due to its open terrain. The southern area of HA is identified as a potential source area, with O_3_ contribution concentrations of 120 μg·m^−3^. Therefore, in addition to local emissions, regional transport plays a crucial role in the distribution of PM_2.5_ and O_3_ pollution in various areas of the FWP due to differences in topographical conditions.

Many studies have also highlighted that the varied impact of regional transport on O_3_ precursor sensitivity from different source areas should be considered when formulating anthropogenic emission control strategies to effectively mitigate O_3_ pollution [65,66]. Given that O_3_-precursor sensitivity (OPS) varies over time, it is imperative to dynamically adjust mitigation strategies by comprehensively considering the variations in OPS in practical implementation.

### 3.3. Dominant Large-Scale Circulation Patterns Driving PM_2.5_ and O_3_ Pollution

In addition to local emissions, the air quality in the FWP is closely linked to the large-scale circulation patterns that influence dispersion and photochemical reaction conditions. Hence, understanding the dominant large-scale circulation patterns driving PM_2.5_ and O_3_ pollution is essential for establishing scientifically effective control measures and improving air quality forecasts. In this study, we counted the number of PM_2.5_ and O_3_ pollution days in the FWP from 2015 to 2022, resulting in 620 days of PM_2.5_ pollution and 544 days of O_3_ pollution. The atmospheric circulation characteristics during the occurrence of polluted weather were analyzed and summarized to identify the circulation background.

#### 3.3.1. Large-Scale Circulation Classification in PM_2.5_

**Figure 4:**
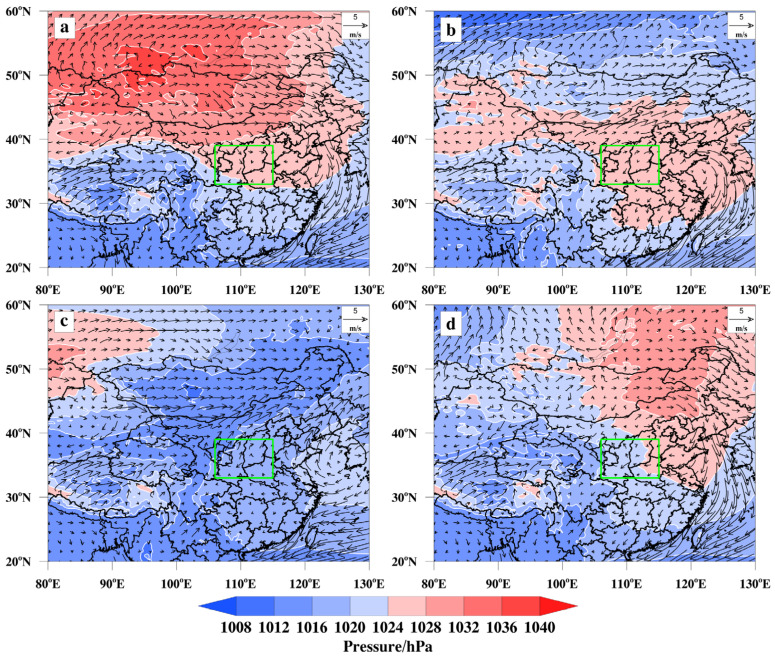
The classification results of large-scale circulation on PM_2.5_ pollution days in the FWP from 2015 to 2022 ((**a**) HPF; (**b**) HPC; (**c**) LPF; (**d**) HPB). The location of FWP plain is highlighted by the green box.

The PCT objective typing method was used to classify the screened sea-level baroclinic fields for PM_2.5_ pollution days in the FWP from 2015 to 2022. The sea-level baroclinic fields were mainly classified into four categories, high-pressure front (HPF), high-pressure control (HPC), low-pressure front (LPF), and high-pressure back (HPB), as shown in Figure 4.

The HPF type appeared most frequently, with 346 pollution days, accounting for 55.81%. A high-pressure system near the Mongolian Plateau moves southeast, causing a decrease in temperature and the appearance of an inversion layer. Additionally, the wind direction changes from southeast to southwest. The FWP’s narrow basin is characterized by mountain ranges in the east and south that obstruct air flow, resulting in the poor diffusion of pollutants and polluted weather. In the second pattern, the FWP and most of the nearby areas are controlled by surface high pressure, accounting for 23.71% of the total pollution days (147 days). This situation is not conducive to the diffusion of PM_2.5_. Additionally, the surface wind speed in the FWP is relatively static, making it difficult for the emitted pollutants to diffuse. This leads to the accumulation of surface PM_2.5_ pollution. In the third pattern, a surface low-pressure system is present from the northeast to the Mongolian Plateau. The LPF type was observed in 68 pollution days, accounting for 10.97%. The FWP is in front of the ground low-pressure system, which causes air flow convergence and leads to increased pollution due to the influence of topography and the inhibited pollutant diffusion. Additionally, the ground wind speed is low, further exacerbating the pollution. The last dominant large-circulation pattern is classified as an HPB type. This type appeared the least number of times, accounting for only 9.52% of the polluted days (59 days). In this pattern, surface cold high pressure is present in the northeast to the north China, with the FWP located behind the high pressure. Simultaneously, cold and wet air, along with pollutants, is brought to the Beijing–Tianjin–Hebei (BTH) region due to the weak easterly air flow near the ground. The static state of the ground wind speed exacerbates the heavy pollution weather in the region. Figure S6 depicts the average PM_2.5_ concentrations observed during four distinct weather processes. Notably, when HPC occurs in the FWP, the average concentration of PM_2.5_ significantly increases, leading to the most severe pollution episodes.

#### 3.3.2. Large-Scale Circulation Classification in O_3_

**Figure 5:**
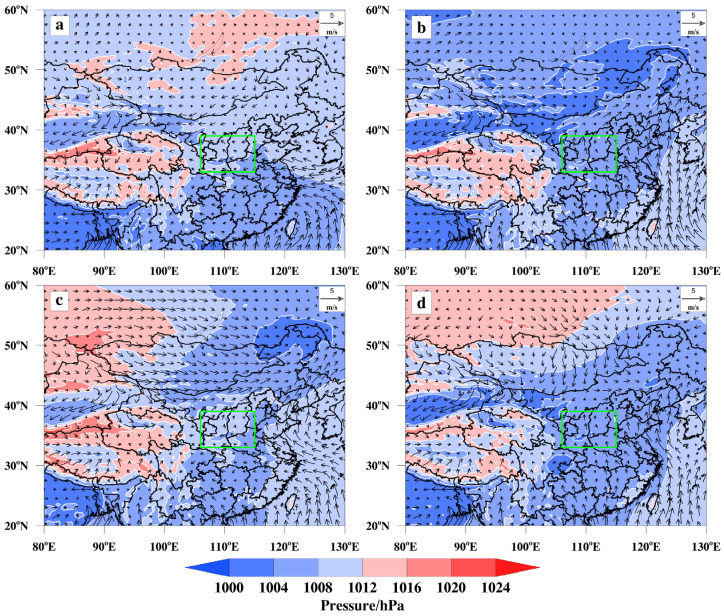
The classification results of large-scale circulation on O_3_ pollution days in the FWP from 2015 to 2022 ((**a**) HPB; (**b**) LPF; (**c**) LTF; (**d**) EPF). The location of FWP plain is highlighted by the green box.

The sea-level baroclinic fields for O_3_ pollution days in the FWP from 2015 to 2022 were classified using the PCT objective typing method. The fields were mainly categorized into four types: high-pressure back (HPB), low-pressure front (LPF), low-pressure trough front (LTF), and equal-pressure field (EPF) (Figure 5).

The most frequently occurring weather pattern associated with high pollution levels is HPB, accounting for 198 polluted days, which represents 36.4% of the total number of days. In this pattern, the FWP is controlled by the surface high-pressure system centered in Lake Baikal. Such a surface high-pressure system leads to a dispersed surface airflow and clear sky, which are conducive to O_3_ production. Due to the narrow basin of FWP, the east and south are blocked by mountain ranges and are influenced by easterly airflow. This hinders the diffusion of pollutants and exacerbates O_3_ pollution. The second most frequently occurring large-scale circulation related to O_3_ pollution is the LPF type, which appeared 149 times, accounting for 27.39%. A surface low-pressure system is centered in Mongolia, with the FWP located in front of it. The FWP is being controlled by a surface high-pressure system, resulting in radiated surface airflow and clear sky. These conditions are conducive to the generation of O_3_. Due to the southerly airflow, pollution was carried northward. However, the transmission of the airflow is obstructed by Lvliang Mountain and Taihang Mountain, which hinder the diffusion of pollution and worsen O_3_ pollution. In the third type, a surface low-pressure cyclone in the northeast and a low-pressure trough extending towards the Mongolian Plateau account for 18.75% of the total, with 102 occurrences. The FWP is located in front of the low-pressure trough and is controlled by surface high pressure. As a result, the surface airflow is dispersed, and the weather is sunny, which is conducive to the generation of O_3_. The southwesterly wind field is unfavorable to pollution diffusion. The EPF type occurred the least frequently, accounting for 17.46% of the total O_3_ polluted days. In this pattern, most parts of China are under the control of a zonal pressure field, resulting in a low-pressure gradient. The presence of low pressure near the FWP, controlled by surface high pressure, is conducive to O_3_ production, while the static wind speed hinders pollution dispersion. Figure S7 displays O_3_ concentration levels throughout four different large-scale circulation. It is observed that O_3_ pollution concentrations reach their peak and result in the most critical pollution conditions during the occurrence of LTF within the surface circulation of the FWP. In summary, when the FWP is under the influence of ground high pressure, the concentration of both PM_2.5_ and O_3_ pollution increases. This is because the ground airflow becomes radicalized and compounded at high altitudes, which hinders the diffusion of pollutants. Simultaneously, in the event of pollution, the horizontal wind speed in the FWP is typically less than 10 m·s^−1^, with low ground wind speed and a static ground state. This unfavorable condition hinders the diffusion of pollutants, leading to their accumulation on the ground and causing pollution.

### 3.4. Source Apportionment

Regional atmospheric transport is a crucial factor influencing the transmission and dispersion of PM_2.5_ and O_3_, while local emissions also play a pivotal role in determining the concentration of pollutants. To gain a deeper understanding of the pollution sources and accurately formulate air pollution prevention and control measures, we utilized PMF to discuss, in detail, the major industries that generate PM_2.5_ and VOCs.

#### 3.4.1. Source Apportionment of PM_2.5_

**Figure 6:**
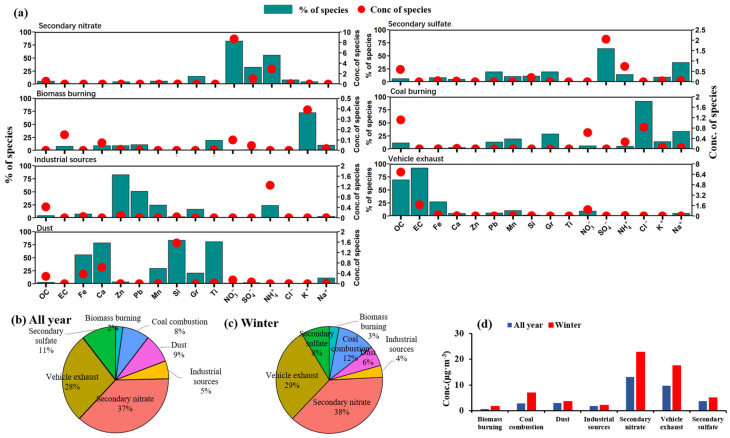
(**a**) Source profiles derived from the PMF model and source contributions to PM_2.5_ concentration. Source contributions to the ambient PM_2.5_ (**b**) all year and (**c**) in winter. (**d**) Factor contribution concentration.

Through multiple iterations of factor number adjustments, we determined that the results with seven factors exhibited good independence and consistency with reality (their contribution concentrations are presented in Figure S8). These factors were identified as secondary nitrates, secondary sulfates, coal combustion, biomass burning, motor vehicle exhausts, fugitive dust, and industrial sources, as depicted in Figure 6. Specifically, Factor 1 was primarily composed of NO_3_^−^ and NH_4_^+^, contributing 82% and 56%, respectively. Since the precursors of these compounds, NO_x_ and NH_3_, can undergo neutralization reactions in the atmosphere to form ammonium nitrate, Factor 1 was identified as secondary nitrate [67]. Factor 2 was dominated by SO_4_^2−^, with a contribution of 64%, primarily originating from the transformation of SO_2_, leading to its classification as a secondary sulfate [67]. Factor 3, characterized by a high content of K^+^ (72%), was recognized as biomass burning due to K^+^ being a typical tracer of this process [68]. Factor 4, dominated by chloride ions (91%), was attributed to coal burning processes, thus identifying it as coal burning [69]. Factor 5, exhibiting high percentages of Zn (83%) and Pb (51%), was identified as an industrial source, primarily associated with industrial processes such as metal smelting [70]. Factor 6 was predominantly composed of EC (92%) and OC (69%), originating primarily from automotive emissions, thus classifying it as motor vehicle exhausts [68]. Finally, Factor 7, which was dominated by elements such as Si (83%), Ti (81%), Ca (79%), and Fe (56%), which are major components of the Earth’s crust, was identified as a fugitive dust source [69]. The particulate matter in XY is primarily derived from secondary nitrates (37%), vehicular emissions (28%), and secondary sulfates (11%). Despite the severe winter pollution, the primary contributors remain largely unchanged. In comparison to the annual average, the contribution of coal combustion sources increases significantly from 8% to 12% during the winter months. This is primarily due to the increased demand for coal heating in the colder winter months. From a concentration perspective, all factors exhibit an increase in winter, with the greatest increase observed in coal combustion, which increased from 2.91 μg·m^−3^ to 6.93 μg·m^−3^. The alterations in dust and industrial sources are relatively minor.

#### 3.4.2. Source Apportionment of VOCs

As shown in Figure S10, we found that the FWP is mainly located in the VOCs-limited regime during the summer, indicating that VOC-focused reductions would be the most effective path to mitigating O_3_ concentrations, whereas NOx reduction would lead to O_3_ rebound.

**Figure 7:**
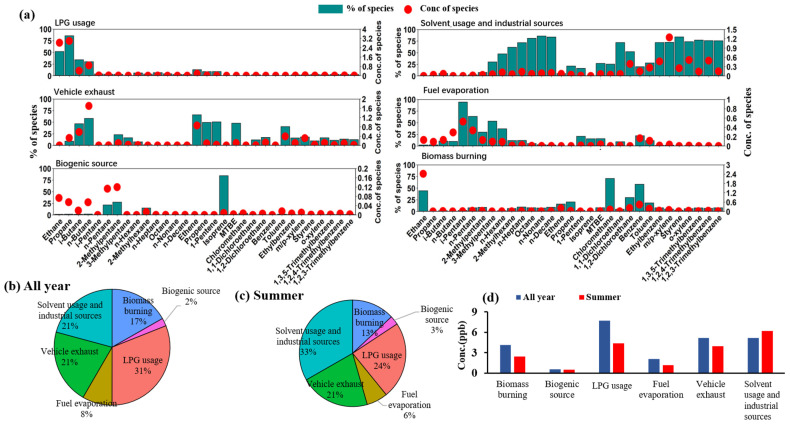
(**a**) Source profiles derived from the PMF model and source contributions to VOC concentration. Source contributions to the ambient VOCs (**b**) all year and (**c**) in summer. (**d**) Factor contribution concentration.

Furthermore, the contribution concentrations of VOCs from various industries to O_3_ were analyzed. Following the application of a series of adjustments to the factor numbers within the PMF model, it was determined that the results obtained with six factors exhibited good independence and conformed to reality. The aforementioned factors included biogenic source, liquefied petroleum gas (LPG) sources, vehicle exhaust, solvent usage and industrial sources, fuel evaporation, and biomass burning. The results are presented in Figure 7. Factor 1, primarily composed of propane (86%), ethane (52%), i-butane (34%), and n-butane (30%), is primarily derived from LPG, thus identifying Factor 1 as LPG sources [71]. Factor 2 exhibits a high contribution from benzene homologues and high-chain alkanes, wherein benzene homologues are commonly used solvents and high-chain alkanes are widely utilized in industrial processes [72], leading to the identification of Factor 2 as solvent usage and industrial sources. Factor 3 exhibits a high contribution from C4–C5 alkanes, such as n-butane and i-butane, as well as C2–C3 olefins; methyl tert-butyl ether (MTBE), a gasoline additive, also contributes significantly. These species are closely related to gasoline vehicle emissions, thus identifying Factor 3 as vehicle exhausts [73]. Factor 4 is characterized by 94% and 64% contributions from i-pentane and n-pentane, respectively, which are typical of oil and gas evaporation, thereby identifying Factor 4 as fuel evaporation [74].

Factor 5, with an 84% contribution from isoprene, primarily originates from plant emissions, leading to the identification of Factor 5 as a biogenic source [75]. While biogenic VOCs can contribute to the formation of O_3_ and secondary organic aerosols [76,77,78,79], the benefits of vegetation in improving air quality through its role as a carbon sink and in reducing other pollutants should not be overlooked. Hence, coordinated deep reductions in anthropogenic VOCs and NOx are a crucial prerequisite for achieving the synergistic control of PM_2.5_ and O_3_. Integrating vegetation-based strategies, such as phytoremediation and urban green zones, alongside technological and industrial interventions, could offer a complementary approach to air pollution control in the FWP. These nature-based solutions can help absorb pollutants, reduce the urban heat island effect, and provide additional benefits to air quality management efforts, particularly in areas with high particulate matter and O_3_ concentrations [80,81,82,83].

Factor 6 exhibits the highest contribution from chloromethane (71%), whose emissions are closely related to biomass combustion. Additionally, benzene (58%) and ethane (44%) also contribute significantly, being associated with incomplete combustion, thus identifying Factor 6 as biomass burning. Overall, VOCs in XY are primarily sourced from LPG (31%), vehicle exhausts (21%), and solvent usage and industrial sources (21%). During the summer, however, the main contributing sources undergo some variations, with solvent use and industrial sources accounting for 33%, LPG accounting for 24%, and vehicle exhausts accounting for 21%. Compared to the annual average, the relative contribution of solvent use and industrial sources increases by 12%, primarily due to the dry and hot conditions in the summer of XY, which results in a larger proportion of volatile solvent sources. The increased contribution of solvent usage and industrial sources to O_3_ concentrations during the summer can be attributed to higher temperatures and dry conditions that enhance the volatilization of industrial solvents. This seasonal pattern aligns with the operational characteristics of industries in the FWP, where certain sectors such as petrochemical manufacturing and vehicle maintenance show higher activity during summer. In terms of concentration changes, the concentrations of all factors, except for solvent use and industrial sources, decrease relatively in the summer.

While VOCs play an important role in O_3_ formation, they do so indirectly through photochemical reactions with NOx under appropriate meteorological conditions. O_3_ formation is a complex process that depends not only on the availability of VOCs and NOx, but also on temperature, solar radiation, and other atmospheric factors. In the FWP during summer, the region tends to be VOC-limited, meaning that reductions in VOC emissions would likely have a more substantial impact on reducing O_3_ concentrations compared to NOx reductions. On the other hand, in addition to local photochemical reactions, O_3_ is also transported into the region through interregional transport, where emissions from nearby or distant areas contribute to elevated O_3_ concentrations [84,85]. Furthermore, stratospheric O_3_ intrusion plays a crucial role in O_3_ levels, especially under the influence of favorable meteorological conditions [86,87].

Furthermore, we have compared the source apportionment results of PM_2.5_ and O_3_ identified from PMF and WRF-CMAQ model in winter and summer, respectively. As seen in Figure S9, the primary sources of PM_2.5_ and O_3_, as assessed by PMF and WRF-CMAQ models, showed similar proportions.

## 4. Conclusions

In this study, we comprehensively utilized hourly pollution data from the FWP spanning the period from 2015 to 2022. Our study fill the gaps in the literature by providing a detailed multi-dimensional analysis of the sources of PM_2.5_ and O_3_ pollution, incorporating both local emissions and regional transport and offering novel insights into the intrinsic connections between local pollution and large-scale circulation.

Temporally, the concentrations of PM_2.5_ are highest during winter, followed by spring, winter, and summer, whereas O_3_ concentration is highest during summer. Spatially, areas with high PM_2.5_ concentrations were found in the middle and west of the FWP, including LF, LY, and XA. O_3_ hotspot areas are mainly observed in the middle of the FWP, specifically in YC and LY. Simultaneously, the concentrations of PM_2.5_ and VOC components were also higher in winter and lower in summer. The results of HYSPLIT indicate that the FWP is primarily impacted by PM_2.5_ originating from the southern Shaanxi air mass and the northwestern air mass during winter. However, in summer, it is mainly influenced by O_3_ from its southern air mass. The major large-scale circulation that contributes to PM_2.5_ pollution in the FWP can be classified as HPF, HPC, LPF, and HPB, which accounts for 55.81%, 23.71%, 10.97%, and 9.52%, respectively. Among them, HPF type is the most of large-scale circulation during periods of severe pollution in the FWP. HPB, LPF, LTF, and EPF were identified as the top four large-scale circulation patterns of O_3_ pollution. The most probable weather type among them is the HPB type, which accounts for 36.40%. Regarding the sources of PM_2.5_, secondary nitrates (37%), vehicle exhausts (28%), and secondary sulfates (11%) are the major contributors. As for VOCs, LPG sources (31%), vehicle exhausts (21%), solvent usage and industrial emissions (21%) are the primary sources.

Given the challenging topographical conditions and energy structure of the FWP, stricter emission control measures are necessary to improve air quality. Furthermore, the dynamic adjustment of O_3_ control strategies based on the varying upwind pollution sources under different large-scale circulation patterns will be crucial for more effective air quality management. This study highlights the fact that using regionally coordinated emission reduction strategies targeting VOCs and NOx is essential for the synergistic control of both PM_2.5_ and O_3_, providing more effective solutions for improving air quality in regions like the FWP. Particularly due much to the changes in OPS, it is critical to dynamically adjust mitigation strategies by comprehensively considering these variations in OPS in practical implementation.

In addition to conventional pollution control measures, integrating nature-based solutions, such as green roofs and urban tree planting, could help absorb pollutants, reduce local O_3_ levels, and mitigate the urban heat island effect in the FWP, particularly in dense urban areas [88,89]. Additionally, we also underscored the importance of characterizing the close connection between large-scale circulation and atmospheric pollution to gain insights into atmospheric processes leading to PM_2.5_ and O_3_ pollution in the FWP and other complex terrain regions worldwide.

## Data Availability

All relevant data are available in the main text.

## Supplementary Materials

The following are available online at https://www.mdpi.com/article/10.3390/toxics13020123/s1, Table S1: Winter air mass trajectory proportion and average PM_2.5_ concentration; Table S2: Summer air mass trajectory proportion and average O_3_ concentration; Figure S1: Seasonal variation in pollution concentrations in the FWP from 2015 to 2022; Figure S2: Spatial distribution of pollutant concentrations in the FWP from 2015 to 2022; Figure S3: The results of cluster analysis of urban airflow trajectories representing (a,c,e) winter and (b,d,f) summer seasons from 2015 to 2022; Figure S4: Distribution of PM_2.5_, NOx and VOC emissions. Figure S5: Potential source contribution analysis of representative urban pollutants in (a,c,e) winter and (b,d,f) summer seasons from 2015 to 2022; Figure S6: PM_2.5_ concentration values of pollutants under four large-scale circulation patterns; Figure S7: O_3_ concentration values of pollutants under four large-scale circulation patterns; Figure S8: The industry-specific contribution concentrations of (a) PM_2.5_ and (b) VOCs; Figure S9: A comparison of the major industrial sources for PM_2.5_ in winter and O_3_ in the summer of 2022 in XA, as identified by PMF and WRF-CMAQ models. Figure S10: The result of OBM for June 2022. References [34,90,91,92,93,94,95,96,97,98,99,100,101,102] are cited in the Supplementary Materials.

## Abbreviations

The following abbreviations are used in this manuscript:

O_3_	Ozone
FWP	Fenwei Plain
HYSPLIT	Single-particle Lagrangian integrated trajectory
PSCF	Potential source contribution function
WPSCF	Weighted potential source contribution function
CWT	Concentration weighted trajectory
WCWT	Weighted concentration weighted trajectory
VOCs	Volatile organic compounds
NOx	Nitrogen oxides
BTH	Beijing-Tianjin-Hebei
YRD	Yangtze River Delta
PRD	Pearl River Delta
NCP	North China Plain
XA	Xi’an
JZ	Jinzhong
LY	Luoyang
XY	Xianyang
APPCAP	Air pollution prevention and control action plan
CNEMC	China National Environmental Monitoring Centre
ECMWF	European Centre for Medium-Range Weather Forecasts
NCEP	National Centers for Environmental Prediction
GDAS	Global Data Assimilation System
NOAA	National Oceanic and Atmospheric Administration
PMF	Positive matrix factorization
PCA	Principal component analysis
CMB	Chemical mass balance
CNEMC	China National Environmental Monitoring Centre
MDA8 O_3_	Daily maximum 8 h average O_3_
PCT	T-model obliquely rotated principal component analysis
BVOCs	Biogenic volatile organic compounds
SOAs	Secondary organic aerosols
MDL	Method detection limit
EF	Error fraction
OVOCs	Oxygenated volatile organic compounds
OPS	O_3_-precursor sensitivity
HPF	High-pressure front
HPC	High-pressure control
LPF	Low-pressure front
HPB	High-pressure back
LTF	Low-pressure trough front
EPF	Equal-pressure field
LPG	Liquefied petroleum gas
MTBE	Methyl tert-butyl ether
