# Supplementary material for: Characterization and Source Apportionment Analysis of PM2.5 and Ozone Pollution over Fenwei Plain, China: Insights from PM2.5 Component and VOC Observations
Source: Toxics. 2025 Feb 6;13(2):123. doi: 10.3390/toxics13020123 (PMC11862001; doi:10.3390/toxics13020123)
Supplement: Supplementary file 1 [file toxics-13-00123-s001.zip › toxics-3422984-supplementary.pdf]

toxics

MDPI

Supplement of

Characterization and Source Apportionment Analysis of $PM_{2.5}$ and Ozone Pollution over Fenwei Plain, China: Insights from $PM_{2.5}$ Component and VOC Observations

Litian Xu [1,], Bo Wang [3,**], Ying Wang [4], Huipeng Zhang [3], Danni Xu [1,2,3,*],Yibing Zhao [4], and Kaihui Zhao [1,3]**

[1] Yunnan Key Laboratory of Meteorological Disasters and Climate Resources in the Greater Mekong Subregion, Yunnan University, Kunming, 650091, China
[2] Information school, Yunnan University of finance and economics, Kunming, 650221, China
[3] Xianyang Environmental Monitoring Station, Shaanxi, 712000, China
[4] Xianyang Meteorological Bureau, Shaanxi, 712000, China;
* Correspondence:zz2331@ynufe.edu.cn.
** These authors cntributed equally to this work.

1. Methods

1.1 Potential Source Contribution Analysis (PSCF and CWT)

The analysis of potential source areas employs both PSCF and CWT methodologies. The PSCF method, developed from conditional probability functions, qualitatively identifies potential pollution sources. It divides the study area into 0.5°×0.5° horizontal grids, quantitatively describing the probability field of source strength for each geographic area. PSCF preliminarily determines the locations of emission sources by integrating air mass trajectories with corresponding values of a certain element. The study area is segmented into $i \times j$ grids, with the PSCF for each grid calculated as follows:

$$P_{ij} = \frac{m_{ij}}{n_{ij}} \quad (1)$$

Where m_{ij} represents the number of pollution trajectories passing through a grid, and n_{ij} denotes the total number of trajectories within that grid. A higher PSCF value for a grid indicates a greater contribution to pollution at receptor points. Since P_{ij} is a conditional probability, the uncertainty in PSCF calculation increases with a higher number of air flow trajectories within a grid, i.e., when the denominator n_{ij} is smaller. To reduce calculation uncertainty, an empirical weighting coefficient W_{ij} is introduced to decrease errors, making the weighted PSCF (WPSCF) equal to WPSCF = W_{ij} × PSCF.

$$W_{ij}\begin{cases} 1.00 \ , & n_{ij} > 80 \\ 0.70 \ , & 20 < n_{ij} \le 80 \\ 0.42 \ , & 10 < n_{ij} \le 20 \\ 0.05 \ , & n_{ij} \le 10 \end{cases} \quad (2)$$

While the PSCF method calculates the proportion of pollution trajectories within a grid, reflecting the degree of pollution contribution to receptor points to some extent, it does not quantify the pollution concentration corresponding to those trajectories. The CWT method calculates the average pollution weight concentration of different grids, thereby quantifying the pollution contribution of different trajectories. The CWT calculation formula is given by:

$$C_{ij} = \frac{\sum_{l=1}^{M} C_l \tau_{ijl}}{\sum_{l=1}^{M} \tau_{ijl}} \quad (3)$$

where C_{ij} is the average weighted concentration of the grid cell, l is the number of trajectories, M is the total number of trajectories, C_l is the concentration of pollutants passing through trajectory l, and τ_{ijl} is the time that trajectory l stays in the grid cell. Similar to the PSCF method, the empirical weighting coefficient W_{ij} is also applicable to the CWT method to reduce the uncertainty in C_{ij}, hence the weighted CWT (WCWT) is calculated as WCWT= CWT × W_{ij}.

1.2 WRF-CMAQ model description

A localized Weather Research and Forecasting (WRF)/Sparse Matrix Operator Kernel Emissions (SMOKE)/Community Multi-scale Air Quality (CMAQ) modeling system was adopted to investigate the $PM_{2.5}$ and O_3 sources during winter and summer 2022 in XA. The Lambert conformal projection centered at 34.25°N, 108.95°E. We set three-nested domain used in this study. The outermost domain 1(D1) covered most of East Asia, Southeast Asia, and the northwestern Pacific, with a grid resolution of 27 km × 27 km, while the middle domain 2 (D2) covered most of Shaanxi Province, with a grid resolution of 9 km × 9 km, and the innermost domain 3 (D3) is the target area in this study, including the Guanzhong basin, with a grid resolution of 3 km × 3 km.

The WRF model (version 3.9) is a meso-scale numerical weather prediction system that is used to simulate the meteorological fields by using the land use data from the Moderate Resolution Imaging Spectroradiometer (MODIS) and meteorological data from the National Centers for Environmental Prediction (NCEP) reanalysis data with 1° ×1° resolution.

The SMOKE model (version 2.1) was used to compute the emissions inputs to the CMAQ from annual county-level emissions inventories. The three domains used the 2016 Multi-resolution Emission Inventory for China (MEIC, http://www.meicmodel.org) with 1° × 1° resolution.

The CMAQ model (version 5.0.2) is a three-dimensional atmospheric chemistry and transport-modeling system. In this study, the CMAQ simulations were represented by the carbon bond mechanism (CB05) for the gas-phase chemistry and the aerosol mechanism (AE05) for the aerosol process. The meteorological fields of the CMAQ were provided by the WRF model. The CMAQ is one-way nested and runs with 18 vertical layers from the surface to 50 hPa.

2. General characteristics of air pollutants in the FWP

Figure S1 displays the seasonal variation of mass concentrations of six conventional pollutants ($PM_{2.5}$, O_3, PM_{10}, SO_2, NO_2, CO) in the FWP from 2015 to 2022. The mass concentrations of $PM_{2.5}$, PM_{10}, SO_2, NO_2, and CO were reached their peak value in winter, followed by spring, autumn and summer. The elevated levels of $PM_{2.5}$ in winter can be attributed to increased combustion for heating purposes [1], coupled with stable meteorological conditions that trap pollutants close to the surface [2]. Lower temperatures, reduced boundary layer heights, and weaker solar radiation during winter contribute to the increased accumulation of $PM_{2.5}$. These conditions facilitate the trapping of pollutants close to the ground, exacerbating air quality issues[3,4]. In contrast, the O_3 concentration exhibits contrasting seasonal variation characteristics, with the peak value occurred in summer, followed by spring and autumn, and the lowest concentrations in winter. The summertime surge in O_3 is primarily due to stronger solar radiation and higher temperatures, which enhance the photochemical reactions between nitrogen oxides (NO_x) and volatile organic compounds (VOCs), leading to O_3 formation[5]. However, relatively lower

levels were observed in winter. This phenomenon is primarily due to the high sensitivity of O_3 concentrations to solar radiation, which is significantly weaker in winter. Additionally, the scattering and absorption effects of sand dust or haze particles suppress the conditions for O_3 formation[6].

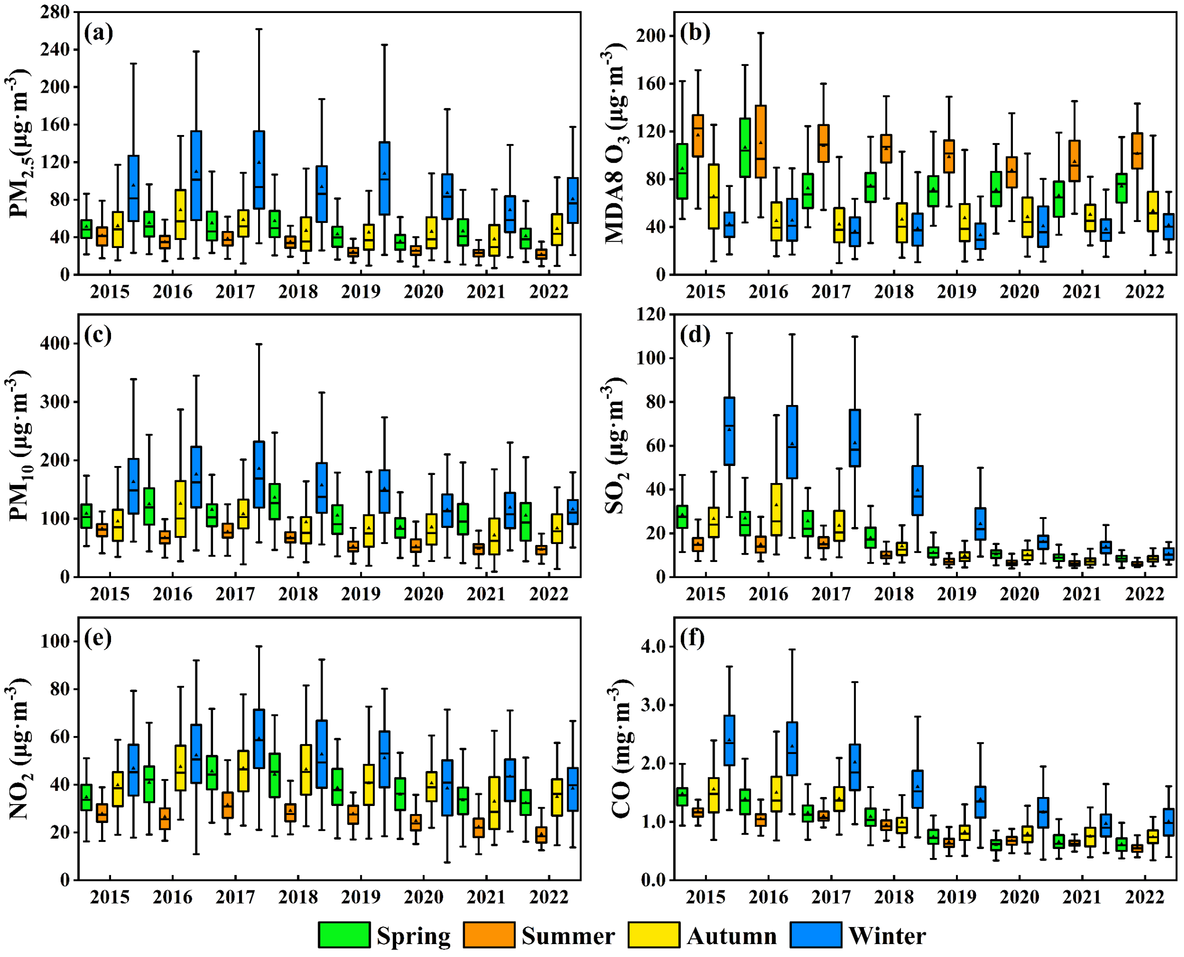

Figure S1. Seasonal variation of pollution concentrations in the FWP from 2015 to 2022. (a) $PM_{2.5}$; (b) O_3; (c) PM_{10}; (d) SO_2; (e) NO_2; (f) CO.

Based on the annual variations in pollutant concentrations, the concentrations of $PM_{2.5}$, PM_{10}, and NO_2 showed a fluctuating upward trend before 2019, decreased in 2020, and then increased in the following year. SO_2 and CO have been decreasing since 2017, with a decrease of 35.24% and 20% on average by 2019. The O_3 concentration remained relatively stable, but began to deteriorate after the 2020 pandemic, with an increase of 8.44% by 2022, indicating that the emission reduction measures taken during the COVID-19 were unfavorable for O_3 pollution control [7]. Concurrently, as the COVID-19 restrictions were gradually lifted in 2021, the winter $PM_{2.5}$ concentration rose from 69.00 $\mu g \cdot m^{-3}$ to 80.66 $\mu g \cdot m^{-3}$ in 2022. It is noteworthy that NO_2 has been decreasing since 2017, with an average annual decrease of 6%, but compared to $PM_{2.5}$ and O_3 pollution, the decrease is not significant, suggesting that current emission reduction strategies are insufficient to achieve coordinated control of $PM_{2.5}$ and O_3 pollution in the FWP.

Figure S2 displays the spatial distribution maps of six conventional pollutants in the FWP from 2015 to 2022. The distribution of $PM_{2.5}$ and O_3 concentrations demonstrates similar characteristics, primarily concentrated in the central region. The formation of O_3 is largely dependent on the reaction of NO_x and VOCs emitted from vehicle exhaust under

sunlight [8], indicating a higher level of pollutant emissions in this area. Conversely, in the XA region, O_3 concentrations are relatively lower, whereas the concentrations of $PM_{2.5}$, PM_{10}, and NO_2 prominently reflect the pollution characteristics of the area, suggesting significant traffic flow and industrial activity. Notably, the distribution patterns of SO_2 and CO exhibit distinct differences from other pollutants. SO_2 is predominantly found in the northern part of the FWP, likely due to emissions from numerous coal-fired industrial facilities in the area, while CO distribution is mainly observed in the LF. These variations reveal the sources and distribution characteristics of different pollutants, which are crucial for a comprehensive understanding and management of regional environmental pollution.

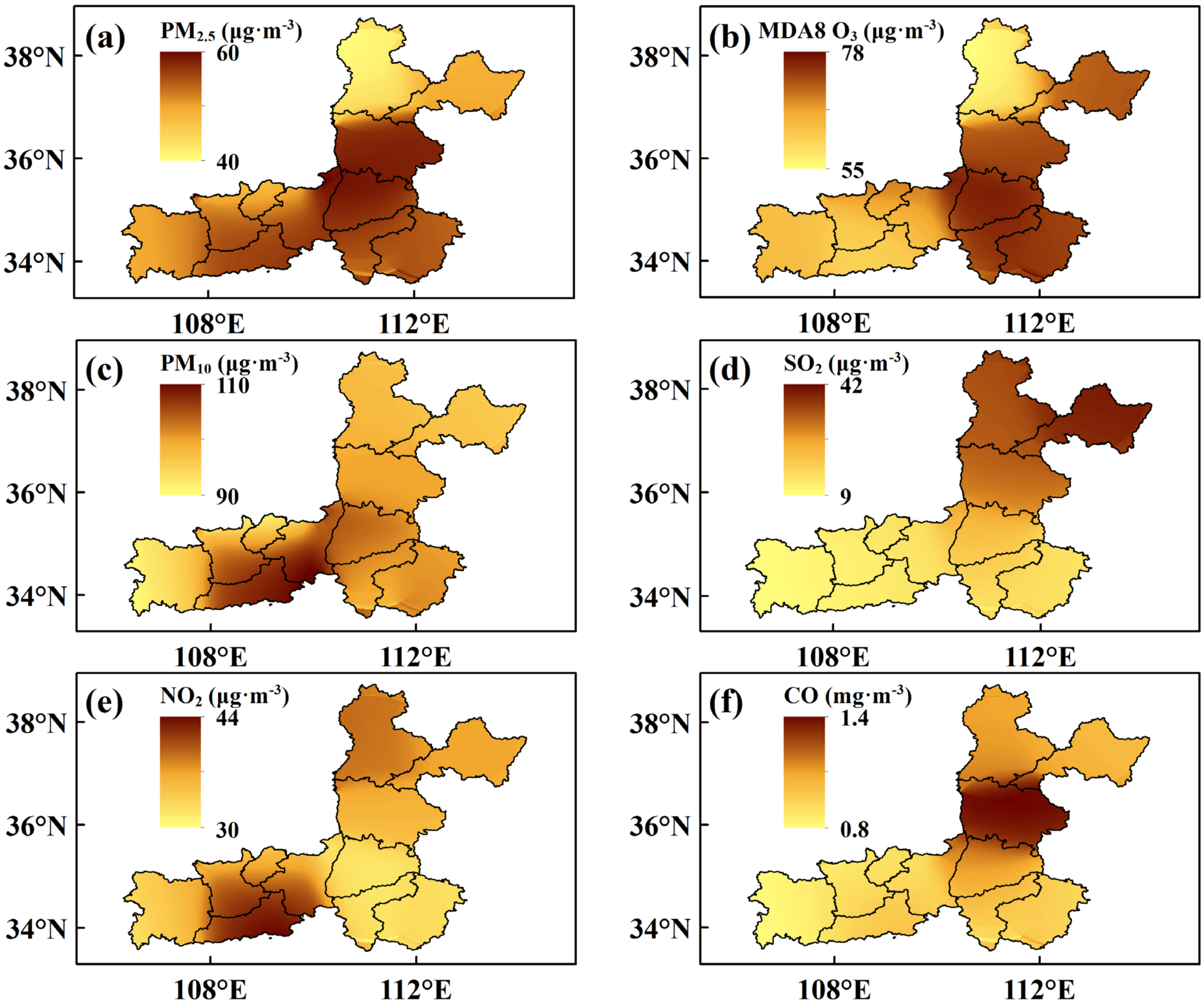

Figure S2. Spatial distribution of pollutant concentrations in the FWP from 2015 to 2022. (a)$PM_{2.5}$; (b)O_3; (c)PM_{10}; (d)SO_2; (e)NO_2 and (f)CO.

3. Analysis of Pollutant Transmission Paths

To examine the pollutant transport pathways in the FWP Plain, we selected XA, JZ, and LY as representative cities from the three most polluted areas of the plain. Between 2015 and 2022, we conducted trajectory simulation and cluster analysis of air mass trajectories during both winter and summer seasons. Figure S3 illustrates the results of the backward trajectory clustering analysis, including the percentage of each trajectory type and the corresponding averaged $PM_{2.5}$ and O_3 concentrations. These quantitative calculations are summarized in Table S1 and Table S2.

Table S1. Winter air mass trajectory proportion and average $PM_{2.5}$ concentration

City	Track	Source	approach	Average $PM_{2.5}$ concentration /($\mu g \cdot m^{-3}$)	Exceedance percentage of track
XA	1	Alxa League, Inner Mongolia	GS、NX	84.89	45.92%
	2	Turpan, XJ	GS、NX	78.34	37.55%
	3	Hanzhong, SN	XA	150.35	87.58%
	4	Kazakhstan	XJ、GS、NX	80.79	34.55%
	5	Zhengzhou, HA	SN	116.89	75.65%
JZ	1	Mongolia	NM、SN	69.62	31.56%
	2	Kazakhstan	Xinjiang、Mongolia、NM、SN	64.46	26.96%
	3	Yanan, SN	LF	97.43	58.97%
	4	Urumqi, XJ	GS、NM、NX	76.82	36.32%
	5	Russia	Mongolia、NM、SN	36.18	8.79%
LY	1	Russia	Mongolia、NM、SN	37.03	9.52%
	2	Mongolia	NM、SN、SX	83.52	40.87%
	3	LF	Zhengzhou(HA)	131.49	81.05%
	4	Kazakhstan	XJ、Mongolia、NX、SN	85.57	40.29%
	5	Ili, XJ	NM、GS、NX、SN	87.90	42.86%

Table S2. Summer air mass trajectory proportion and average O_3 concentration

City	Track	Source	approach	Average O_3 concentration /($\mu g \cdot m^{-3}$)	Exceedance percentage of track
XA	1	BJ	XA	93.64	38.06%
	2	Mongolia	NM、SN	95.76	42.27%
	3	Kazakhstan	XJ、GS、NX	111.59	55.24%
	4	Shangqiu, HA	XJ、GS、NX	86.19	31.68%
	5	Yulin, GX	GZ, CQ	79.02	27.98%
JZ	1	BTH	HE	106.11	48.54%
	2	Mongolia	NM、SN	97.94	45.20%
	3	Kazakhstan	XJ、Mongoli、NM、SN	103.39	53.57%
	4	Yanan, SN	GS、NM、NX	107.00	52.81%
	5	Bozhou, AH	Zhengzhou(HA)	114.00	57.89%
LY	1	BTH	HE, HA	107.07	50.14%
	2	Mongolia	NM、SN、SX	118.06	60.23%
	3	Yueyang, HB	HB	111.74	55.94%
	4	NM	SN、SX	108.89	52.99%
	5	Yellow Sea	JS, HA	102.72	45.84%

During winter, the FWP is affected by Siberian cold and high pressure. The three representative cities of XA, JZ, and LY are influenced by air masses from the northwest, accounting for 60.70%, 73.79%, and 63.66% of the total number of trajectories, respectively. XA, located in the western part of the FWP, has a trajectory of 28.41% from the southern part of the neighborhood. This air mass corresponds to the highest $PM_{2.5}$ concentration of 150.35 $\mu g \cdot m^{-3}$, with an exceedance rate of 87.58%. The air mass trajectory's short transport distance suggests a stable atmospheric environment with sufficient water vapor, conducive to the generation and accumulation of $PM_{2.5}$ pollution. This indicates that despite fewer trajectories from the southwest direction, it contributes significantly to $PM_{2.5}$ exceedance events. JZ located in the north of the FWP, primarily has its air mass trajectories

originating from the northern area of SN to its west, accounting for 26.21% of the total trajectories. This trajectory corresponds to the highest $PM_{2.5}$ concentration of 97.43 μg·m^{-3}, with an exceedance rate of 58.97%. This trajectory passes through the northern region of SN, characterized by loess plateaus and hills, which are prone to significant dust production. This phenomenon exacerbates downstream $PM_{2.5}$ pollution levels. LY is situated in the middle of the FWP. The air mass trajectory from LF in the northern part of its neighborhood accounted for 36.34%, which corresponded to the highest $PM_{2.5}$ concentration of 131.49 μg·m^{-3}, with an exceedance rate of 81.05%. LF discharges a significant amount of pollutants due to increased industrial activities and heavy industrial structures. The $PM_{2.5}$ pollution is further exacerbated as it passes through areas such as the northern area of HA, indicating that LY is primarily affected by long-distance pollution transport from the north.

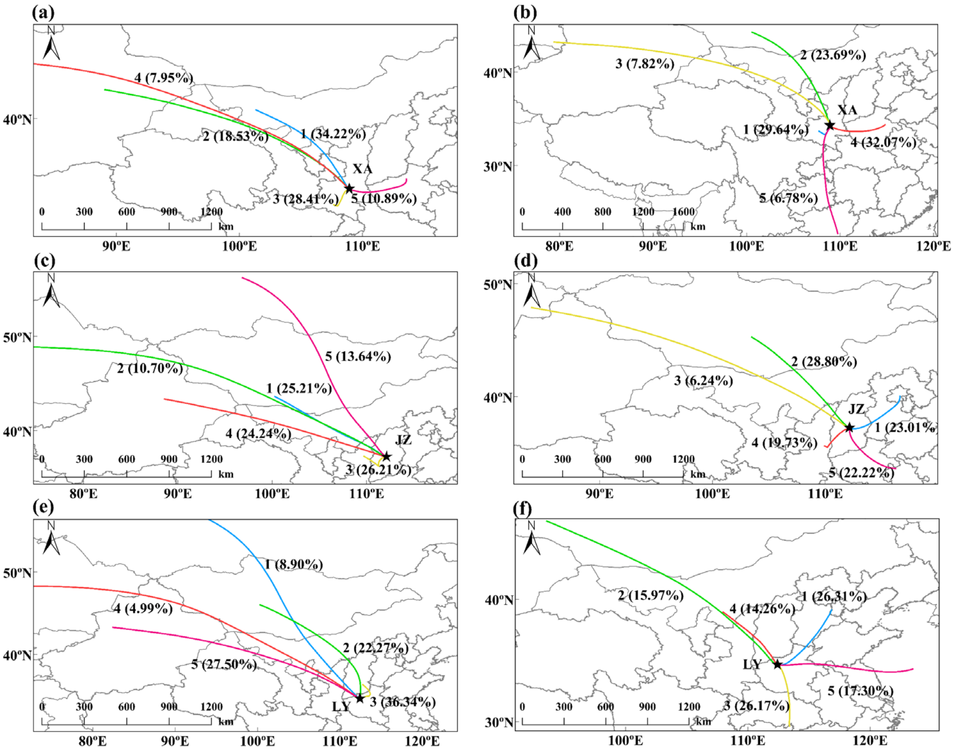

Figure S3. The results of cluster analysis of urban airflow trajectories representing (a, c, e) winter and (b, d, f) summer seasons from 2015 to 2022.

As shown in Figure S3b, S4d, and S4f, during summer, the FWP is affected by summer monsoon, and the three representative cities of XA, JZ, and LY are influenced by the regional transport from southeast, accounting for 38.85%, 45.23%, and 69.78% of the total number of trajectories, respectively. XA is affected by regional from the eastern area of HA, accounting for 32.07% of the total number of tracks (Figure S3b). However, its O_3 concentration remains in a low level of 86.19 μg·m^{-3}. Tracks 2 and 3 from Mongolia and Kazakhstan represent a relatively small proportion of the total number of tracks, accounting for 23.69% and 7.82% respectively. However, due to the continental climate in the regions of Mongolia and Kazakhstan, strong summer solar radiation fosters O_3 photochemical reactions, resulting in relatively high average O_3 concentrations, with average concentrations of 95.76 μg·m^{-3} and 111.59 μg·m^{-3} respectively. This suggests that XA is susceptible to long-distance transport of O_3 from Northwest China in summer. JZ is also susceptible to air mass track5 from the northern area of AH, which corresponds to the highest O_3

concentration of 114.00 μg·m^{-3}, with an exceedance rate of 57.89% (Figure S3d). JZ is susceptible to O_3 pollution from neighboring transmission in summer, as indicated by its susceptibility to cross-regional transport from SN and BTH. The transport from the northern area of HN dominates $PM_{2.5}$ level in LY due to its gently undulating terrain (Figure S3f). This trajectory has a share of 26.17% and corresponds to an average O_3 concentration of 111.74 μg·m^{-3}, with an exceedance rate of 55.94%. The proportion of track 2 originating from Mongolia is only 15.97%, while its track has the highest average concentration at 118.06 μg·m^{-3}, with an exceedance rate of 60.23%. LY is susceptible to long-range O_3 pollution, as well as pollution transported from the Yellow Sea and BTH.

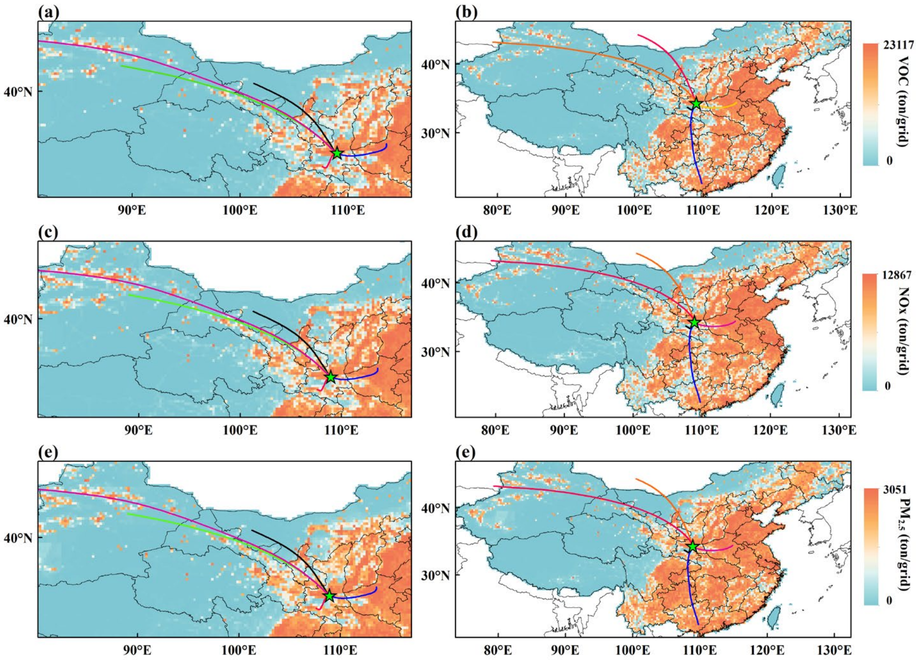

Figure S4. Distribution of $PM_{2.5}$, NOx and VOC emissions.

4. Analysis of Potential Source Areas

Identifying potential source areas of $PM_{2.5}$ and O_3 pollution can provide effective scientific support for regional joint prevention and control efforts. Here, we used the PSCF method to identify the potential sources in the three representative cities from 2015 to 2022. The standard value of $PM_{2.5}$ was set to 75 μg·m^{-3} and the standard value of MDA8 O_3 was set to 100 μg·m^{-3}, based on Ambient air quality standards (GB 3095-2012). Higher WPSCF values indicate that the area is more likely to be a potential source area for the polluted receptor cities.

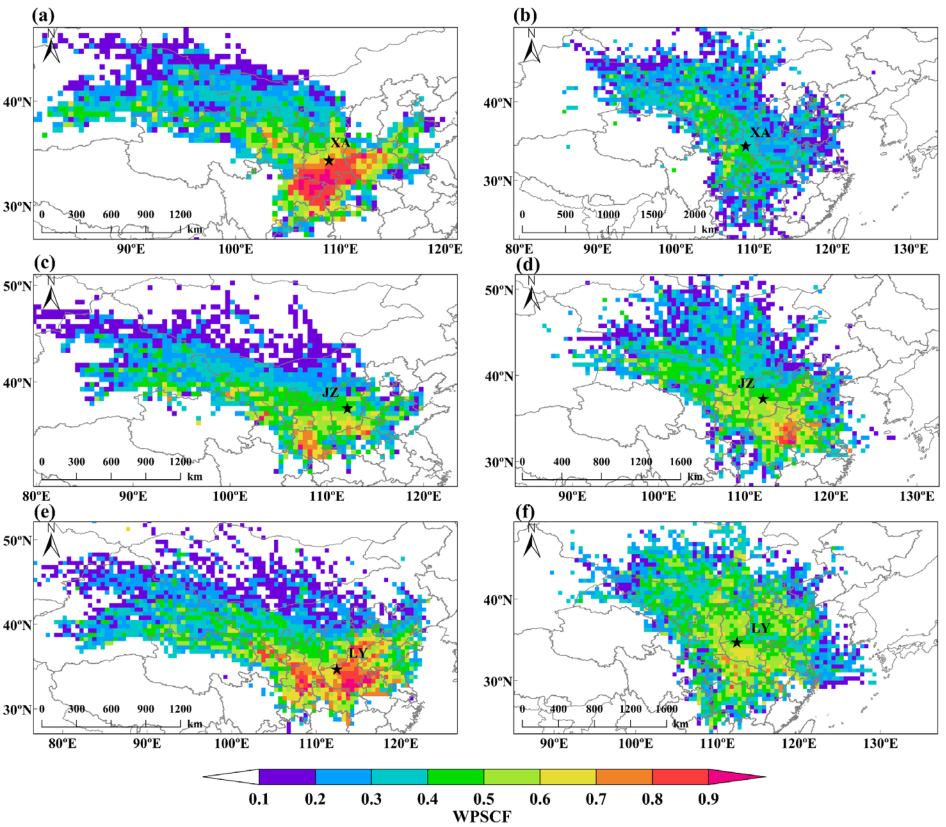

Figure S5. Potential source contribution analysis of representative urban pollutants in (a, c, e) winter and (b, d, f) summer seasons from 2015 to 2022. The map was created using MeteoInfo software (version 3.7.9, http://meteothink.org). MeteoInfo is widely used for creating weighted analysis maps from HYSPLIT Model.

Figure S4a, S4c, and S4e illustrate the analysis of $PM_{2.5}$ pollution source areas in representative cities during winter. The potential pollution source areas of the three cities exhibit a westnorth-southeast band distribution, centered over the GS. However, the WPSCF value is below 0.4, indicating a low impact of pollution on the representative cities. The potential source areas of XA are primarily located in southern SN and western HA. The WPSCF values in these areas exceed 0.7, making them the primary source of $PM_{2.5}$ pollution. The potential source areas of JZ are mainly located in the southern SN and the northern area of HA, with WPSCF values exceeding 0.7. The potential source area of LY is relatively extensive. The majority of pollution sources are situated on the eastern and western sides, particularly in southern SN and HA. Their WPSCF values exceed 0.7, indicating that the main influence on the concentration of $PM_{2.5}$ in LY is primarily from the transport from neighboring areas.

Figure S4b, S4e, and S4f illustrate the analysis of potential source area of O_3 pollution in representative cities during summer. Unlike the distribution characteristics of potential $PM_{2.5}$ pollution source areas, O_3 pollution source areas exhibit a patchy distribution pattern. Western areas of XA, such as the eastern area of SC and the western area of GS, have WPSCF values ranging from 0.4 to 0.7, indicating that these areas are the primary potential source areas of O3 in XA. The potential areas in JZ are mainly located in the eastern part of HA, with WPSCF values above 0.7. Furthermore, the YRD is also an important potential pollution source area for JZ, with a central WPSCF value above 0.7, indicating long-range transport of pollutants from YRD to the northern FWP.

5. Large-scale circulation classification in $PM_{2.5}$

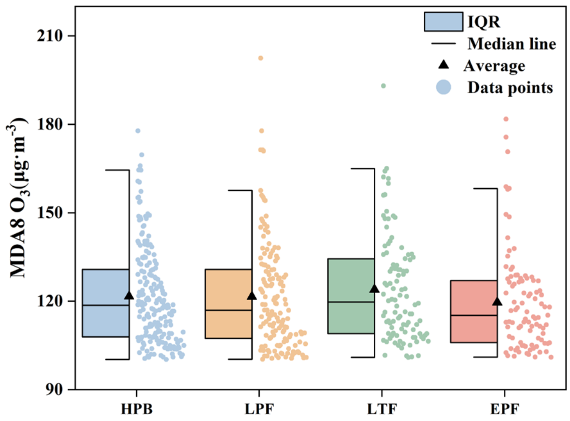

Figure S6. $PM_{2.5}$ concentration values of pollutants under four large-scale circulation patterns

6. Large-scale circulation classification in O_3

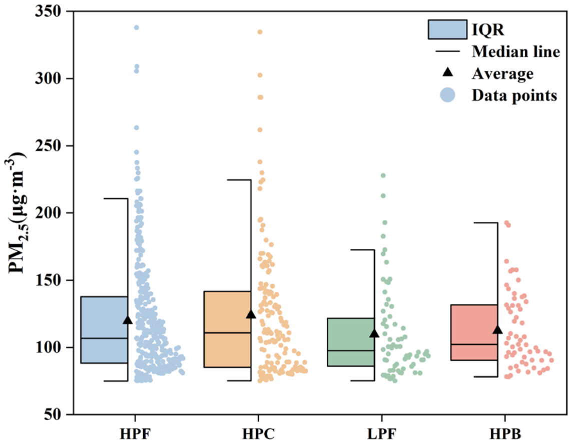

Figure S7. O_3 concentration values of pollutants under four large-scale circulation patterns

7. Source apportionment

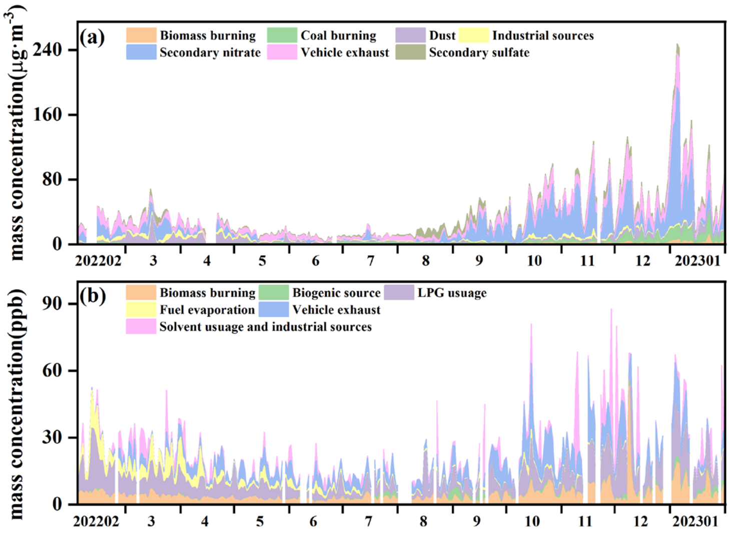

Figure S8. The industry-specific contribution concentrations of (a) $PM_{2.5}$ and (b) VOCs.

8. WRF-CMAQ

We have compared the source apportionment results of $PM_{2.5}$ and O_3 identified from PMF and WRF-CMAQ model in winter and summer, respectively. As seen in Figure S9, the primary sources of $PM_{2.5}$ and O_3, as assessed by PMF and WRF-CMAQ models, showed similar proportions. Industrial source accounted for the largest proportion (40% for PMF vs. 39.1% for WRF-CMAQ of $PM_{2.5}$, 37% for PMF vs. 39.6% for WRF-CMAQ of O_3), followed by the transportation source (31% for PMF vs. 21.8% for WRF-CMAQ of $PM_{2.5}$, 28% for PMF vs. 29.7% for WRF-CMAQ of O_3).

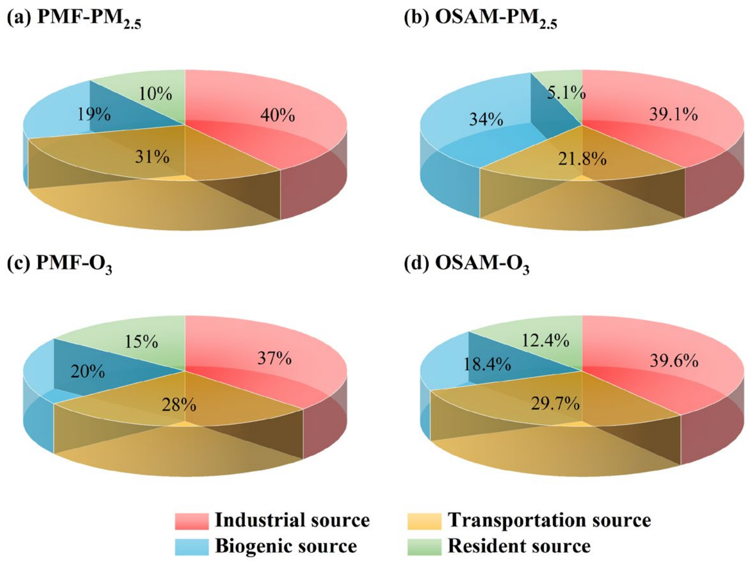

Figure S9. A comparison of the major industrial sources for $PM_{2.5}$ in winter and O_3 in the summer of 2022 in XA, as identified by PMF and WRF-CMAQ models.

9. O_3-precursor sensitivity (OPS) in the FWP

To efficiently control O_3, the O_3-precursor sensitivity (OPS) must first be revealed. For a given region, OPS can be divided into VOC-limited, NOx-limited, and transitional regimes. Generally, OPS is more likely to be VOC-limited in urban areas and NOx-limited rural areas [9,10].

To address the reviewer's concern, we identify the OPS in FWP by using the Observation-Based Model (OBM). OBM is a data-driven approach that analyzes the formation of air pollutants, by utilizing observational data. The input data typically include ground-based monitoring station concentrations, meteorological parameters (e.g., temperature, humidity, wind speed, and direction), as well as VOCs concentrations. These data are processed and used to generate the Empirical Kinetics Modeling Approach (EKMA) curve, which illustrates the relationship between O3 formation and precursor concentrations. By analyzing the EKMA curve, OBM helps quantify the contributions of NOx-limited and VOC-limited to O3 formation and provides insights into its source mechanisms[11,12].

As shown in Figure S10, we found that the FWP is mainly located in the VOCs-limited regime during the summer, indicating that VOC-focused reductions would be the most effective path to mitigating O_3 concentrations, whereas NOx reduction would lead to O_3 rebound [12–14].

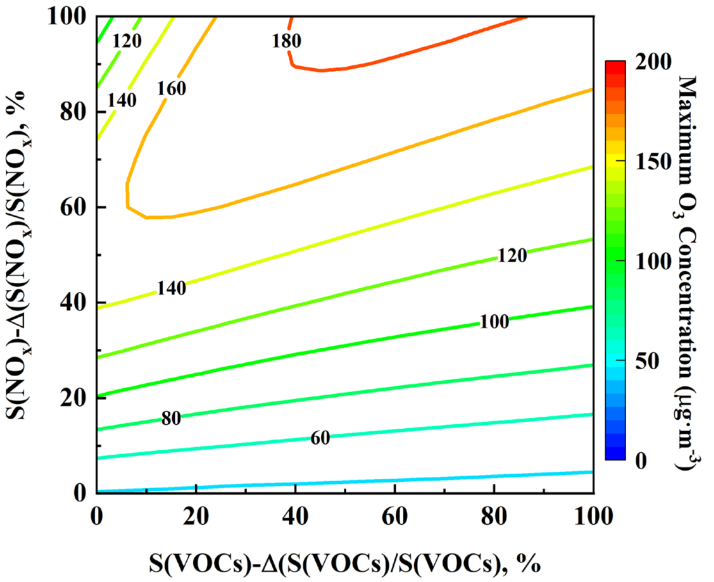

Figure S10. O_3 isopleth diagram at XY station in June 2022.

References

1. Sun, Y.; Zhou, X.; Wai, K.; Yuan, Q.; Xu, Z.; Zhou, S.; Qi, Q.; Wang, W. Simultaneous Measurement of Particulate and Gaseous Pollutants in an Urban City in North China Plain during the Heating Period: Implication of Source Contribution. *Atmospheric Research* **2013**, *134*, 24–34, doi:10.1016/j.atmosres.2013.07.011.
2. Zhang, Y.-L.; Cao, F. Fine Particulate Matter (PM2.5) in China at a City Level. *Sci Rep* **2015**, *5*, 14884, doi:10.1038/srep14884.
3. Li, Q.; Li, X.; Li, H. Factors Influencing PM2.5 Concentrations in the Beijing–Tianjin–Hebei Urban Agglomeration Using a Geographical and Temporal Weighted Regression Model. *Atmosphere* **2022**, *13*, 407, doi:10.3390/atmos13030407.
4. Xiao, Q.; Ma, Z.; Li, S.; Liu, Y. The Impact of Winter Heating on Air Pollution in China. *PLoS ONE* **2015**, *10*, e0117311, doi:10.1371/journal.pone.0117311.
5. Yin, Z.; Cao, B.; Wang, H. Dominant Patterns of Summer Ozone Pollution in Eastern China and Associated Atmospheric Circulations. *Atmos. Chem. Phys.* **2019**, *19*, 13933–13943, doi:10.5194/acp-19-13933-2019.
6. Wang, Z.; Fang, C. Spatial-Temporal Characteristics and Determinants of PM2.5 in the Bohai Rim Urban Agglomeration. *Chemosphere* **2016**, *148*, 148–162, doi:10.1016/j.chemosphere.2015.12.118.
7. Zhang, Q.; Zhang, Y.; Zhang, W.; Wei, P.; Wang, Q.; Li, Z.; Shen, Z. Magnitude and Origins of Severe Urban Air Contaminants in China during the COVID-19 Lockdown: A Comprehensive Analysis. *Atmospheric Research* **2023**, *295*, 106999, doi:10.1016/j.atmosres.2023.106999.
8. Ren, H.H.; Cheng, Y.; Wu, F.; Gu, Z.L.; Cao, J.J.; Huang, Y.; Xue, Y.G.; Cui, L.; Zhang, Y.W.; Chow, J.C.; et al. Spatiotemporal Characteristics of Ozone and the Formation Sensitivity over the Fenwei Plain. *Science of The Total Environment* **2023**, *881*, 163369, doi:10.1016/j.scitotenv.2023.163369.
9. Wang, W.; Van Der A, R.; Ding, J.; Van Weele, M.; Cheng, T. Spatial and Temporal Changes of the Ozone Sensitivity in China Based on Satellite and Ground-Based Observations. *Atmos. Chem. Phys.* **2021**, *21*, 7253–7269, doi:10.5194/acp-21-7253-2021.
10. Ren, J.; Guo, F.; Xie, S. Diagnosing Ozone–NO_x –VOC Sensitivity and Revealing Causes of Ozone Increases in China Based on 2013–2021 Satellite Retrievals. *Atmos. Chem. Phys.* **2022**, *22*, 15035–15047, doi:10.5194/acp-22-15035-2022.
11. Chu, W.; Li, H.; Ji, Y.; Zhang, X.; Xue, L.; Gao, J.; An, C. Research on Ozone Formation Sensitivity Based on Observational Methods: Development History, Methodology, and Application and Prospects in China. *Journal of Environmental Sciences* **2024**, *138*, 543–560, doi:10.1016/j.jes.2023.02.052.
12. Wang, W.; Fang, H.; Zhang, Y.; Ding, Y.; Hua, F.; Wu, T.; Yan, Y. Characterizing Sources and Ozone Formations of Summertime Volatile Organic Compounds Observed in a Medium-Sized City in Yangtze River Delta Region. *Chemosphere* **2023**, *328*, 138609, doi:10.1016/j.chemosphere.2023.138609.
13. Xu, D.; Yuan, Z.; Wang, M.; Zhao, K.; Liu, X.; Duan, Y.; Fu, Q.; Wang, Q.; Jing, S.; Wang, H.; et al. Multi-Factor Reconciliation of Discrepancies in Ozone-Precursor Sensitivity Retrieved from Observation- and Emission-Based Models. *Environment International* **2022**, *158*, 106952, doi:10.1016/j.envint.2021.106952.

14. Liu, Z.; Hu, K.; Zhang, K.; Zhu, S.; Wang, M.; Li, L. VOCs Sources and Roles in O3 Formation in the Central Yangtze River Delta Region of China. *Atmospheric Environment* **2023**, *302*, 119755, doi:10.1016/j.atmosenv.2023.119755.
